# Impact of dyadic practice on the clinical self-efficacy and empathy of nursing students

**DOI:** 10.1186/s12912-022-01171-y

**Published:** 2023-01-09

**Authors:** Maryam Kamali, Shirin Hasanvand, Parastou Kordestani-Moghadam, Farzad Ebrahimzadeh, Mitra Amini

**Affiliations:** 1 Student Research Committee, Lorestan University of Medical Sciences, Khorramabad, Iran; 2 Social Determinants of Health Research Center, School of Nursing and Midwifery, Lorestan University of Medical Sciences, Khorramabad, Iran; 3 Nutritional Health Research Center, School of Health and Nutrition, Lorestan University of Medical Sciences, Khorramabad, Iran; 4 Clinical Education Research Center, Shiraz University of Medical Sciences, Shiraz, Iran

**Keywords:** Peer learning, Empathy, Self-Efficacy, Nursing, Students

## Abstract

**Background:**

Dyadic practice of learners creates supportive learning. So far, few studies have investigated the impact of this approach on students’ empathy and self-efficacy. This study aimed to investigate the effect of dyadic practice on nursing students’ clinical self-efficacy and empathy.

**Methods:**

This study was based on a pretest-posttest randomized group from September to December 2018. All the junior nursing students (*n* = 44) were divided into intervention (*n* = 22) and control groups (*n* = 22) using stratified random sampling. The intervention group was trained for 6 days as student dyads, while the control group was under the supervision of an instructor and worked individually. The students’ levels of empathy and self-efficacy were evaluated on the first day (pretest) and the last day (post-test) by The Self-Efficacy in Clinical Performance Scale and Mehrabian and Epstein empathy scale. The data were analyzed using the SPSS software by Fisher’s exact test, Mann-Whitney test, independent t-test paired t-test, Wilcoxon signed-rank, and Analysis of covariance.

**Results:**

Dyadic practice increased empathy in the intervention group compared to the control group (*P* < 0.001). The adjusted mean of total empathy in the intervention group was 21.1 degrees higher than the adjusted mean of total empathy in the control group. However, no significant differences were found between the two groups in clinical self-efficacy (*P* = 0.762).

**Conclusions:**

The employment of this approach seems helpful in creating an empathic atmosphere. However, further studies are required to prove the effectiveness of this method on self-efficacy.

## Background

One of the primary purposes of medical sciences universities is empathy and its development [1]. According to Heggestad (2018), one moral and professional necessity for a nurse is to identify and fulfilling the patient’s needs, which requires empathy [2]. As a critical element in taking care of patients, empathy increases patients’ satisfaction, trust, and compliance with treatment [3–5]. Empathy has been mentioned as an integral component to help with social skills such as teamwork. Also, empathy by developing students’ resilience reduces their learning burnout [6].

Despite the significant role of empathy, the evidence shows low levels of empathy in nursing students. In the study by Williams et al. (2016) investigating students’ level of empathy in different disciplines of nursing, midwifery, and emergency health, the midwifery students’ level of empathy was higher than that of the other two groups [7]. The study by Ferri et al. (2017) revealed a minor decrease in nursing students’ level of empathy during the study process [8]. Similarly, a recent descriptive study in Iran showed that nursing students’ empathy levels decreased significantly in the fourth year compared to the first year [9].

Empathy is closely related to several factors, including self-efficacy. In a study, empathy was correlated with self-esteem, interpersonal relationships, and self-efficacy [10]. Self-efficacy has been regarded as an important motivational factor [11] and influential in students’ professional development [12]. Brannagan et al. (2013) emphasize the importance of self-efficacy assessment regarding students’ clinical skills to evaluate the effectiveness of educational methods [13]. According to Bandura (1994), perceived self-efficacy is defined as people’s beliefs in their capabilities to create designated levels of performance that exercise influence over events affecting their lives [14]. In nursing, self-efficacy is defined as nurses’ beliefs in their abilities to apply their professional expertise based on their achieved knowledge when taking care of patients [15, 16].

The development of self-efficacy is essential in nursing [17]. Skill competence requires students’ belief in their ability to use their knowledge and skills correctly [18, 19]. Self-efficacy strengthens nursing students’ self-esteem through stress management and compatibility, self-control, and concentration resulting in a successful function. Also, Self-efficacy represents one of the determinants of career preferences and is the strongest predictor of professional identity and fostering resilience [20, 21].

According to Kim (2018), teachers are responsible for increasing students’ empathy through education [10]. Nursing instructors employ different teaching methods to increase students’ levels of empathy and self-efficacy, one of which is peer learning [1]. Empathy develops in peer learning, and individuals gain information from their peers more easily and share their secrets [22]. As Lopez-Mondéjar and Pastor (2017) revealed, collaborative learning improves emotional and social skills such as empathy and decisiveness in the classroom [23]. In addition, few authors have found that peer learning affects self-efficacy as a critical element in professional development in nursing [12, 24], and evidence shows that one of the four sources of self-efficacy beliefs is vicarious experiences through observing peers [25].

Black and MacKenzie suggested two vertical and horizontal models in peer learning [26]. In the horizontal model, students receive support from their peers at the same level [27]. Horizontal or reciprocal peer tutoring is also called paired or dyadic learning [28, 29]. Dyad learning is a subcategory of reciprocal peer-assisted learning in which pairs of same-level students share learning responsibilities [30]. In this method, students of the same levels are engaged in different educational experiences; they take turns playing the roles of a tutor and a tutee [24]. As Ott and Succheralli (2015) described in the sense of student clinical partner dyads, student dyads are the students who take care of the same patient on the same shift, with the same instructor, and in the same place [28, 29, 31]. This model is a targeted educational method to encourage and increase self-confidence [31]. According to Tolsgaard et al. (2014), dyadic practice improves the efficiency of clinical skills. The results of their phenomenological research indicated that the student’s level of self-efficacy increased as a result of interactions with their peers [32]. In addition, some studies indicated a decrease in the student dyads’ level of anxiety and stress and increased confidence in their abilities [33, 34].

However, the results concerning the impact of peer learning on students’ self-efficacy are very different and occasionally paradoxical, which requires further research. Furthermore, there are limited published data on the impact of peer learning on empathy [35]. Most national and international studies have focused on the vertical peer learning model [36], whereas few studies have been conducted on the impact of collaborative learning with reciprocal peer tutoring and its efficiency in clinical environments [37]. Therefore, regarding students’ positive views on this approach [38], the economic aspect of the approach [39], and a few studies with occasionally paradoxical results, the present study aims to investigate the impact of dyadic practice on clinical self-efficacy and empathy of Iranian’ nursing students.

## Methods

### Study design and setting

This study was a randomized controlled trial conducted from September to December 2018 at one of the Nursing Schools of Lorestan University of Medical Sciences (Western Iran).

### Participants and recruitments

The sample size was calculated based on the following formula.

$${\frac{{\left(z1-\frac{\alpha\ }{2}+z1-\beta \right)}^2\kern0.75em \left(2s\ \right)}{d^2}}^2$$

The sample size was 20 people in each group. Due to falling samples, the final sample size of 22 people for each group was considered. The whole enumeration method was used according to the population (44 people). The details of estimating the sample size were as follows:

$$\alpha \to 0/05\ {Z}_1\frac{\alpha }{2}=1.96$$

$$\upbeta =0/1\to {\textrm{Z}}_{1-\upbeta}=1.28$$

$$S\approx \frac{R}{6}=\frac{185-37}{6}=24.67=25$$

$$d=25$$

S = Standard deviation of the self-efficacy questionnaire score in each group.

d = The minimum difference between the mean self-efficacy scores in the two groups is important for the researchers.

**Fig. 1:**
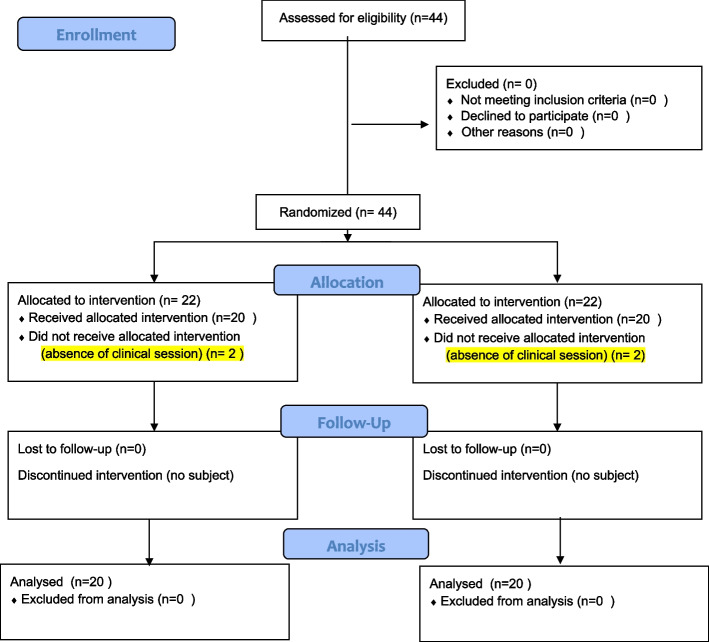
Flow chart of the study

In Iran, the Bachelor of Nursing is 4-years, full-time, under a standard curriculum. This study chose 44 third-year (5th term), undergraduate nursing students, by the whole enumeration. They completed a theoretical course on Adults and Older Adults Nursing II in the second semester of the academic year 2017–2018. Adults and Older Adults Nursing II, including, Respiratory, Cardiovascular, and Kidney Disorders, is presented in the second year (4th term). Then, the clinical course is presented in the third year (5th term). The participants (*N* = 44) were divided into six groups of 7–8 members by stratified random allocation. Three groups were used as interventions, and the other was used as the control. The permuted block randomization and a random numbers table to randomly allocate students to two groups (control or intervention) were used in each stratum. Part of a table of random numbers was selected. The numbers in a column were read from top to bottom. If the number read was between 0 and 4, the person was assigned to the control group. Instead, if a number was between 5 and 9, the subject was assigned to the intervention group. Out of 44 participants, 40 people completed the study (Fig. 1).

The internship courses lasted four consecutive weeks (Two days a week). The subjects selected were as follows: passing the theoretical course of cardiovascular nursing, the desire to participate in the study and undergraduate students of the fifth semester. The students who did not intend to continue their participation nor had more than one absence were excluded. The inclusion criteria for the instructors were having clinical work experience in the cardiac unit and having at least 2 years of teaching experience.

In this study, the researchers actively participated in the course’s instructional design and were responsible for coordinating the program.

### Intervention

Before data collection, all students explained the model’s research purpose and the intervention groups in the orientation session. The clinical education sessions were from 7:30 am to 13:30. The participants were asked to complete the research instruments on the first day of the internship(pretests) and the last day (post-test). During the first week, all students were asked to take care of a patient (with myocardial infarction or angina pectoris) individually based on the nursing process and under an instructor’s supervision. The reason for choosing the desired cases was that, firstly, most of the patients hospitalized in the heart unit were the mentioned patients. Second, to compare the control and intervention groups, it would be better to define the cases more precisely and narrowly. The students’ performances were evaluated at the end of the second day. In the intervention groups, the students who had achieved the highest grades were selected and asked to choose their dyad from the other students.

#### Intervention group

In intervention groups, the learners performed comprehensive care of the patient in each shift, including a thorough assessment of the patient in a 6-hour shift, writing and implementing a care plan according to the nursing process, recording the report based on the problem-oriented record format, and executing medication orders. Also, activities such as diagnostic procedures, visits or rounds with the doctor, and post-discharge training were included (Table 1). For the daily assessment of learners, periodic oral and written reports were delivered by learners based on the appropriate format to the clinical instructor, and the necessary feedback was provided. Based on the competencies expected of the learners in each session, their performance was scored. The educational content related to the nursing management of cardiac patients was taken from Brunner & Suddarth’s Textbook of Medical-Surgical Nursing, NANDA nursing diagnosis list (2015–2017), and Bates’ Guide to Physical Examination and History Taking (2017). The content validity of the management of patients with myocardial infarction and angina pectoris was evaluated by three faculty (Ph.D. in nursing with experience in teaching courses related to cardiovascular diseases) and two clinical nurses (with experience in the heart ward and coronary care unit) and one Cardiologist.

**Table 1:** Nursing management in a patient with myocardial infarction or angina pectoris

Component	Description
**Initial assessment**	Obtain a health history, and review patient records Perform a comprehensive physical assessment to detect complications and changes in the patient’s status Monitoring vital signs Assess for chest pain, shortness of breath, dyspnea, tachypnea, crackles, nausea, vomiting, decreased urinary output, and assess IV sites frequently.
**Actual and potential nursing diagnosis**	Presenting the NANDA nursing diagnosis list (2015–2017) to students, including Acute pain, Activity Intolerance, Fear/Anxiety, Risk for decreased cardiac output, Risk for ineffective tissue perfusion, Risk of excess fluid volume, Deficient knowledge of other possible nursing care plans.
**Planning and Goals**	Write nursing care plans, short-term and long-term goals, and Set priorities and writing outcomes.
**Nursing Interventions**	Selecting and performing nursing interventions, Documenting care, Giving verbal reports to a supervisor
**Evaluation**	Determine if goals have been met and re-evaluate as necessary and document.
**Discharge and Home Care Guidelines**	Write a discharge care plan

### Design of student dyad

The dyad accepted the responsibility for taking care of a patient assessment, medications, documentation, teaching the patient, listening to reports for their patient, assisting peers in all shifts, attending diagnostic procedures, physician/nurse rounding, and respecting the learning process of their peers. Initially, they were given guidance in designing the care plan via a supervisor, but their supervision of the students gradually diminished. Both peers had to formulate and implement a care plan after the patient’s initial evaluation, determining their needs and prioritizing them. One of the students acted as the senior during one shift and trained the other. The students changed their roles during the next shift, and the same cycle was repeated for 3 weeks) second to the fourth week (. During the second week, each caring dyad once experienced the role of senior. With the supervision and assistance of the instructor, they would adjust the patient care plan together, and the patient care continued until the end of the shift under the general supervision of the supervisor. However, during the third and fourth weeks, when each care dyad had the senior role twice, they prepared a care plan. Then the instructor informed the patient’s care by the care dyad, and the care process continued with minimal supervision.

### Control group

The control group’s performance was similar to the intervention group’s. The only difference between the two groups was that learners took care of the patient in the control group individually by supervision for all 4 weeks.

All the groups were provided with two supervisors who had good teaching and clinical experiences. Before beginning the study, a one-day workshop was held to ensure the trainers’ same level of training to familiarize them with the model, description of model elements, and taking care of patients according to the nursing process as well as familiarize them with dyad formation, the technique of peer learning, and the peers’ roles, lastly how to manage process flow.

### Assessment tools

#### The Self-Efficacy in Clinical Performance (SECP) scale

SECP scale was composed of two parts. The first part included the students’ demographic characteristics and the second part consisted of four dimensions (assessment, planning of the care plan, implementation, and evaluation of the care plan) with 37 items based on a scale of 0–100, from completely no confidence to complete confidence (Not at all sure:0–20%, not sure:30–40%, relatively confident:60–50%, confident:80–70%, and completely confident:100–80%). The highest and lowest scores of the instrument are 37 and 185, respectively. The Cronbach’s alpha was 0.96 for overall SECP and 0.90–0.92 for the subscales. The results of the test-retest (r = 0.94) suggest the scale’s stability [40].

#### Mehrabian and Epstein’s Emotional Empathic Tendency Scale (EETS) (1972)

Mehrabian and Epstein were used to measure the learner’ level of empathy. It consisted of 33 items and seven subscales of reactive empathy, verbal empathy, collaborative empathy, emotional susceptibility, emotional stability, empathy with others, and control. The items are designed on a 5-point Likert scale (from Completely agree (1) to completely disagree (5)). The lowest and highest scores are 33 and 165. Using the test-retest method, Mehrabian and Epstein reported a reliability coefficient of 0.84 and a Cronbach’s alpha of 0.88 [41].

### Data analysis

All the analyses were conducted using IBM SPSS Statistics for Windows. A one-sample k-s test was used to check the normality of the data. In cases where the *p*-value was less than 0.2, nonparametric tests such as Mann-Whitney were used. An independent t-test was also used for cases where the *p*-value was greater than or equal to 0.2. Wilcoxon signed-rank and paired t-tests were used for within-group comparison (before and after). The effect of baseline scores will be adjusted using an analysis of covariance. The *p*-value below 0.5 (*P* < 0.05) was considered statistically significant.

## Results

Finally, 40 people completed the study. The two groups were similar in sociodemographic characteristics (Table 2). However, both groups differed in sex, and the study adjusted the effects. There is no significant difference between the baseline self-efficacy scores of the control and intervention groups before the intervention.

**Table 2:** Comparison between two groups in terms of sociodemographic characteristics

Groups Variables		Control	Intervention Group	***P***-Value
		Frequency (%)^a^	Frequency (%)^a^	
Age (year)	18–23	15(0.75%)	16(80.0%)	0.465
	24–29	15(25.0%)	3(15.0%)	
	≤30	0(0.0%)	1(5.00%)	
Gender	Female	13(65.0%)	8(40.0%)	0.205
	Male	7(35.0%)	12(60.0%)	
Marital Status	Married	3(15.0%)	2(10.0%)	> 0.999
	Single	17(85.0%)	18(90.0%)	
Residence Status	Non-Dormitory	8(40.0%)	10(50.0%)	0.751
	Dormitory	12(60.0%)	10(50.0%)	
Average Passed Courses	Below 15	9(45.0%)	8(40.0%)	> 0.999
	15 Or More	11(55.0%)	12(60.0%)	

^a^For qualitative variables, frequency (percentage) and median (quartile range) have been used for quantitative variables

In the control group, there was no significance regarding the dimensions of assessment, diagnosis and planning, and implementation before and after the study. However, there was a significant change in the dimension of evaluation (*p* = 0.012) and the total self-efficacy score (*p* = 0.044). There was a significant change in the intervention group’s dimensions and the total self-efficacy score (*p* = 0.001). When the pretest scores were controlled statistically, the results indicated no significant differences between self-efficacy scores’ mean change between the two groups. According to the results, the difference between the two groups in terms of average score change after adjusting the baseline self-efficacy score was in different dimensions and the total self-efficacy score (Table 3).

**Table 3:** Comparison between the changes in the scores of self-efficacy and its dimensions in the intervention and control groups

Dimensions of self-efficacy	Control group					Intervention group				***p***-value	
		Before	After	Change	*p-value	Before	After	Change	*p-value	Model 1**	Model 2***
**Assessment**	Mean (SD)	39.2 (12.26)	41.35 (6.61)	2.15 (11.83)	0.126	33.2 (5.43)	40.15 (6.67)	6.95 (6.83)	**0.001	0.124	0.885
**Nursing diagnosis and planning**	Mean SD	24.45 (7.58)	27.7 (5.12)	3.25 (8.95)	0.052	23.65 (5.06)	29.3 (4.66)	5.65 (5.41)	**0.001	0.311	0.375
**Implementation**	Mean (SD)	32.3 (8.37)	34.6 (6.32)	2.3 (8.26)	0.088	31.05 (5.4)	34.45 (5.33)	3.4 (6.04)	**0.025	0.634	0.907
**Evaluation**	Mean (SD)	17.15 (5.24)	20.2 (3.9)	3.05 (6.01)	**0.012	15.95 (35.2)	19.2 (2.87)	3.25 (3.24)	**0.001	0.897	0.515
**Total self-efficacy**	Mean (SD)	113.1 (26.93)	123.85 (19.09)	10.75 (27.95)	**0.044	103.85 (14.89)	123.1 (16.64)	19.25 (16.1)	**0.001	0.246	0.762

* Wilcoxon test for intra-group comparison at the beginning and end of the study

**In Model 1, the Mann-Whitney test was used without adjusting the base values

***In Model 2, Analysis of covariance was used to adjust the base values

Results revealed no significant change in the overall empathy scale and its dimensions in the control group before and after the study (*p* = 0.888). However, there was a significant change in the intervention group between the total empathy score and its dimensions before and after the intervention (*p* < 0.001). The results indicated that, when the pretest scores and gender were controlled statistically, there was a significant difference between the mean changes of the empathy of the intervention and control groups (*P* < 0.001) (Table 4); that the adjusted mean change of empathy scores in the intervention group was 20.544 points higher than the control group.

**Table 4:** Comparison between the changes in the scores of empathy and its dimensions in two groups

Dimensions	Control group					Intervention group				***p***-value	
		Before	After	Change	***p***-value*	Before	After	Change	***P***-value*	Model 1**	Model 2***
**Reactive**	Mean (SD)	2.77 (0.47)	2.6 (0.44)	0.17 (0.75)	0.999	3. (0.339)	3.36(0.297)	0.35 (0.290)	0.012	0.001	0.001
**Verbal**	Mean (SD)	2.2 (0.3)	2.28 (0.37)	0.12 (0.43)	0.999	2.56 (0.33)	3.58 (0.43)	1.02 (0.6)	0. 421	0.001	0.001
**Collaborative**	Mean (SD)	2.7 (0.3)	2.75 (0.59)	0.04 (0.63)	0.999	2.67 (0.31)	3.29 (0.47)	0.62 (0.63)	0.021	0.003	0.001
**Emotional susceptibility**	Mean (SD)	2.77 (0.63)	2.89 (0.55)	0.92 (0.77)	0.999	2.66 (0.47)	3.42 (0.38)	0.76 (0.63)	0.036	0.001	0.001
**Emotional stability**	Mean (SD)	2.8 (0.55)	0.58 (0.41)	0.21 (0.69)	0.999	2.86 (0.5)	3.25 (0.54)	0.38 (0.67)	0.045	0.004	0.001
**Empathy with others**	Mean (SD)	2.71 (0.42)	2.85 (0.5)	0.13 (0.66)	0.999	2.69 (0.47)	3.2 4 (0.58)	0.55 (0.78)	0.022	0.001	0.001
**Control**	Mean (SD)	2.67 (0.71)	2.77 (0.71)	1. (0.91)	0.999	2.45 (0.79)	3.45 (0.72)	1. (0.96)	0.001	0.036	0.001
**Total**	Mean (SD)	89.95 (6.77)	90.3 (8.20)	0.35 (12.08)	0.888	89.65 (5.68)	111.1 (5.19)	21.45 (9.34)	*p* < 0.001	*p* < 0.001	*p* < 0.001

*Wilcoxon test for intra-group comparison at the beginning and end of the study

**In Model 1, an independent T-test was used without adjusting the base values

***In Model 2, covariance analysis was used to adjust the base values

## Discussion

Contrary to expectations, the current study found that the dyadic practice approach did not increase self-efficacy in the intervention group compared to the control group. This finding is contrary to that of Pålsson et al. [42], who found that dyadic practice increases self-efficacy in the intervention group. In their study, a preceptor was allocated to the dyads, and an instructor supervised them a few times parallel to the preceptor, whereas, in the current study, the two groups were guided by only an instructor. The study results by Parchebafieh et al. (2016) suggested the positive impact of peer learning on self-efficacy. In the mentioned study, the control and intervention groups received clinical training from an instructor and peers (near peers, not dyad), respectively. The results indicated that peer learning increased the students’ sense of clinical self-efficacy. The authors argue that difference in level is essential for peers to transfer their knowledge*,* which is valid for near peers who are always at a higher level. However*,* dyad peers are academically equal and slightly inexperienced [43].

In another study aiming to investigate the impact of mentorship on nursing students’ stress levels*,* sense of belonging*,* self-efficacy*,* and loneliness*,* the findings indicated a decrease in stress levels and loneliness and an increase in self-efficacy and sense of belonging [44]. Griffin and Griffin investigated the impact of collaborative learning on students’ self-efficacy [31]. The findings indicated that this method increases self-efficacy. According to them, this and some educational methods are probably more beneficial than other methods. According to the qualitative study by Moore et al. (2021), dyad practice improves students’ self-efficacy and increases educational efficiency, especially in the first clinical years [30].

Moreover, skill competence requires self-efficacy. Some studies confirmed the effect of peer learning on increasing clinical skills and clinical self-efficacy. In a semi-experimental study, Bahar et al. (2022) showed the positive effect of peer learning on the psychomotor skills of nursing students [45]. Abbott et al. (2021) compared dyad versus individual simulation-based training on performance. Dyad practicing caused similar performance versus solo training [34].

Although this result differs from some studies mentioned above, it is consistent with Brannagan et al. They used the method of near-peer learning in their study [13]. The literature shows that one of the most common methods to increase self-efficacy is the behavior of peer modeling and imitation, which positively impacts learning outcomes. However, Bandura [14] argues that vicarious experiences through effort and perseverance can lead to self-efficacy beliefs [46]. It seems that, in this study, the time was probably insufficient to form self-efficacy beliefs.

A possible explanation for this is that the selection of the dyads based on evaluating their level of knowledge may influence the results. One of the challenges of modeling research is determining a suitable peer. In the current study, dyad formation was carried out only based on an initial cognitive assessment. Artino points out that not all the models are equally effective, and these models have a higher impact on self-efficacy when understood as homogeneous, enthusiastic, and reliable models [47]. One study on factors determining a mentor’s relationships points to the level of satisfaction, depth of relationships, and similarities between the mentor and the mentee [35]. In another study, the criterion for forming a pair was the score obtained by the dyad from the empathy score [48].

Contrary to self-efficacy, the results suggested an increase in the level of empathy after the intervention. Based on the evidence, interactive programs lead to the development of interpersonal skills [49]. The results of López-Mondéjar and Pastor, in which a collaborative learning classroom was used, indicated increased empathy among the members [23]. According to Quince et al.^,^ a hidden curriculum probably influences the students’ level of empathy [50]. Like the present study, Ginsberg et al. (2021) used peer tutoring for third-year nursing students. Pairs were determined by the initial assessment of students using the Jefferson Scale of Physician Empathy for Healthcare Science Students. In this way, the student with the highest empathy score formed a pair with the student who obtained the next highest score, and the following pairs were formed similarly. This study showed a significant improvement in empathy in the intervention group. They suggested peer learning as an effective method for developing empathy in nursing students [48].

The development of empathy is influenced by factors such as genetics and imitation from early childhood [32]. Imitation is an essential human learning habit that plays a crucial role in most human skills, behaviors, and interests [51].

According to Tolsgaard et al.^,^ the neurophysiological basis of learning occurs through observation and imitation by the mechanism of mirror neurons [32]. Observing others’ actions and imitating these actions lead to changes in the system of mirror neurons in the brain. These changes lead to learning, empathy, and kinesthetic skills [52, 53]. These neurons transfer the sensory information obtained from others’ kinesthetic actions to a similar action so that observers themselves can imitate the same kind of action [54].

Furthermore, another mechanism of the nervous system is neuroplasticity [55]. Many studies demonstrate the creation of new intramural synopses during the learning process, which changes and removes the older inactive synapses [56]. To make efficient neuroplasticity in students, dyad learning must be implemented for a long time with more stability so that the learner can develop higher levels of skills and apply them independently. The limitation is that the current study was conducted for three consecutive weeks. In addition, it was not possible to provide performance independently. It is recommended that interventions for self-efficacy last at least 6–8 weeks and students be provided with independent educational tasks. In this study, the sample size was small. It is suggested that the study be performed with a higher sample size. Also, there was a probability of sharing the learning process between them.

In summary, applying a peer learning approach, as opposed to individual learning, increases students’ levels of empathy. Empathy increases patients ‘satisfaction and adherence to treatment and reduces anxiety. Considering the growing trend of the nursing student population, the lack of nursing educators on the one hand, and the decrease in support of clinical nurses from students due to the workload, it is suggested to use this approach as an alternative approach in the clinical setting. However, further studies with a longer duration are required due to the ineffectiveness of the intervention on students’ self-efficacy. According to the clinical self-efficacy outcome results, clinical instructors must pay enough attention to how to form a pair and the stability of peer relationships in using this approach. Other future directions for research include: comparing the role of dyad practice versus individual learning on students’ resilience and interpersonal communication skills.

## Data Availability

The datasets generated and analyzed during the current study are not publicly available due to confidentiality but from the corresponding author at a reasonable request.
